# Pain Management in Autosomal Dominant Polycystic Kidney Disease: Clinical Challenges and a Stepwise Algorithmic Approach

**DOI:** 10.34067/KID.0000000907

**Published:** 2025-07-07

**Authors:** Abdul Hamid Borghol, Fadi George Munairdjy Debeh, Ahmad Ghanem, Marie Therese Bou Antoun, Vineetha Rangarajan, Jonathan Mina, Mohamed Hassanein, Lyle W. Baker, Sahil Gupta, Shennen A. Mao, Christy L. Hunt, Marie C. Hogan, Michael A. Mao, Fouad T. Chebib

**Affiliations:** 1 Division of Nephrology and Hypertension, Department of Medicine, Mayo Clinic, Jacksonville, Florida; 2 Mayo Clinic Florida PKD Center of Excellence, Jacksonville, Florida; 3 Department of Internal Medicine, Staten Island University Hospital, Northwell Health, Staten Island, New York; 4 Division of Nephrology, Kidney and Urology Center, Alexandria, Egypt; 5 Department of Pain Medicine, Mayo Clinic, Jacksonville, Florida; 6 Department of Transplantation, Mayo Clinic, Jacksonville, Florida; 7 Division of Pain Medicine, Department of Anesthesiology and Perioperative Medicine, Mayo Clinic, Rochester, Minnesota; 8 Division of Nephrology and Hypertension, Department of Medicine, Mayo Clinic, Rochester, Minnesota

**Keywords:** ADPKD, BP, cell signaling, CKD, chronic kidney failure, chronic renal disease, chronic renal failure, cystic kidney, kidney volume, liver cysts

## Abstract

Autosomal dominant polycystic kidney disease (ADPKD) is the most common genetic kidney disorder. It is primarily caused by pathogenic variants in the *PKD1* or *PKD2* genes. This leads to the development of numerous kidney cysts, which can result in kidney enlargement and progression to kidney failure. Pain is a common symptom in ADPKD and can negatively affect quality of life (QOL). This pain is often due to the continuous growth of kidney and liver cysts or associated cystic complications. We present a case of a 29-year-old woman with ADPKD who experienced chronic, refractory right-sided flank pain that significantly affected her QOL. Her pain persisted despite taking daily multimodal analgesics and undergoing multiple invasive interventions. She had an unusual asymmetric disease with the right kidney accounting for only 24% of her kidney function. After exhausting all other pain control strategies, she underwent right nephrectomy and partial hepatectomy, which led to substantial improvement in pain and QOL. This review describes the causes, manifestations, and management strategies for abdominal and/or flank pain in ADPKD, including a practical stepwise algorithm to guide clinicians in managing pain and improve QOL of patients with ADPKD. Pain in ADPKD can either be acute or chronic and can lead to significant physical and psychologic distress. Effective pain management in ADPKD requires a multidisciplinary approach, incorporating both nonpharmacologic and pharmacologic interventions such as gabapentin or tolvaptan in select cases. Interventions considered in ADPKD pain control include cyst aspiration with sclerotherapy, celiac plexus blockade, spinal cord stimulation, renal denervation, or nephrectomy.

## Introduction

Autosomal dominant polycystic kidney disease (ADPKD) is the most common genetic kidney disease and is predominantly caused by pathogenic variants in the *PKD1* or *PKD2* genes.^1,2^ ADPKD is characterized by the formation of numerous cysts, leading to kidney enlargement and kidney failure.^3,4^ It presents with a wide range of renal and extrarenal manifestations.^5^ Pain affects 60% of patients and is often related to kidney and/or liver enlargement, cyst complications, and compression of nearby structures.^6–9^ Patients with ADPKD commonly report pain in the flank or back (71%) or the abdomen (61%),^10,11^ typically described as a dull ache, sensation of fullness, and/or stabbing cramping pain.^12^ Beyond physical discomfort, chronic pain in ADPKD significantly affects patients' social and occupational function, leading to anxiety, depression, sleep disturbance, social isolation, reduced productivity at work, and a diminished quality of life (QOL).^10,13–15^

We present the case of a 29-year-old patient with ADPKD whose pain caused considerable distress and was refractory to a series of interventions culminating in the need for unilateral nephrectomy and partial hepatectomy. We also provide a comprehensive review of pain in ADPKD, including its etiology, pharmacologic, and nonpharmacologic management strategies. Finally, a comprehensive algorithm is proposed for the complex management of pain in ADPKD.

## Case Presentation: Initial Presentation and Diagnostic Evaluation

### Medical History

A 29-year-old woman with ADPKD, a history of recurrent kidney cyst ruptures, and migraine headaches presented for evaluation of chronic right-sided flank pain. She had a strong family history of ADPKD including her mother, who was diagnosed at age 40 years, and maternal grandmother. Her surgical history was significant for two C-sections at age 23 and 25 years, with the second one being complicated by preeclampsia at the end of the third trimester. She is married with two kids and is a lifelong nonsmoker. She was diagnosed with ADPKD at age 11 years when she developed right-sided abdominal pain. Her symptoms were initially misdiagnosed as appendicitis, requiring several days of hospitalization for acute pain control. From ages 11 to 28 years, the patient experienced intermittent chronic pain with occasional exacerbations, including two to three acute flank pain episodes annually, requiring daily pain management.

At age 28 years, she experienced right-sided pain, fever, and gross hematuria due to *Escherichia coli* urinary tract infection and a presumed cyst rupture, requiring intravenous antibiotics. Abdominal computed tomography (CT) revealed right kidney enlargement with multiple cysts, with a few scattered cysts in the left kidney, as well as cysts in the liver and pancreas. No signs of obstruction were observed. Her right kidney volume was 709 ml, while her left kidney volume was 218 ml. eGFR by creatinine and cystatin C was 65 and 93 ml/min per 1.73 m^2^, respectively. Genetic testing using clinical grade next-generation sequencing panel revealed a *PKD2* pathogenic variant (c.2614C>T; p/Arg872*).

### Initial Assessment

**Figure 1:**
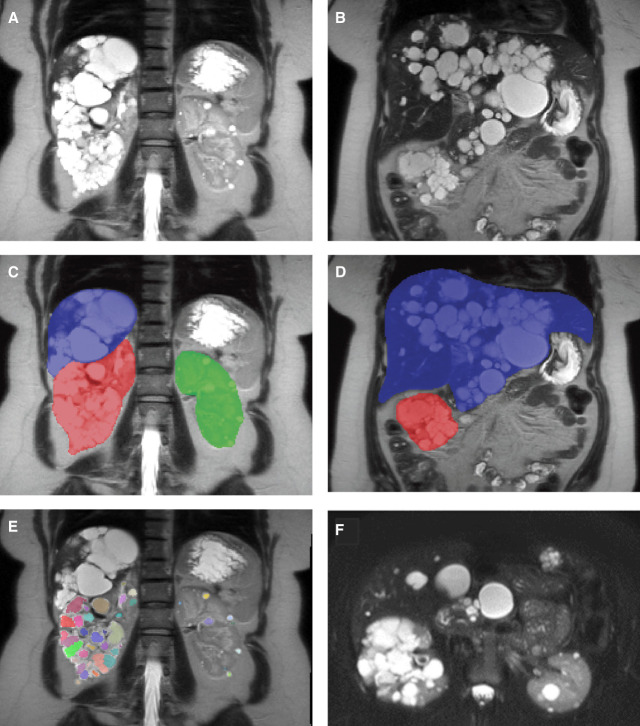
**Imaging representation of the kidney and liver involvement in our patient.** T2 MRI images of the patient (A) coronal section of the right and left kidneys, (B) coronal section showing the extent of liver involvement, (C) coronal sections with the (RKV: 631.7 ml) and (LKV: 218 ml), (D) coronal section showing the (TLV: 2615 ml) corresponding to a height-adjusted TLV of 1656.8 ml, (E) coronal section of the right and left kidneys with cyst segmentation, and (F) axial sections of the right kidney, left kidney, and the liver. Magnetic resonance images and predicted kidney segmentations were processed through a cyst segmentation model to identify and label individual cysts. Using Python and the PyRadiomics library, the model computes key imaging biomarkers, including total cyst count, total cyst volume, cyst-parenchymal surface area, and additional advanced metrics.^16–18^ LKV, left kidney volume; MRI, magnetic resonance imaging; RKV, right kidney volume; TLV, total liver volume.

Her chronic right flank pain localized along the midaxillary line and radiated to the costovertebral area. Initial conservative therapies, including physical therapy, massage, acupuncture, and pain medications (acetaminophen/tramadol), were ineffective. Pain relief required hydrocodone-acetaminophen (10–325 mg), which reduced her pain from 5–6/10 to 3/10, with occasional exacerbations (7/10). Tolvaptan was not recommended due to the asymmetric cystic phenotype (Mayo Imaging Class-2A; Figure 1), which is not considered a risk of rapid progression. Transcutaneous electrical nerve stimulation (TENS) provided minimal relief with subsequent recommendation to start gabapentin 100 mg twice daily. The lack of large/dominant cysts made her case unsuitable for cyst aspiration or sclerotherapy.

## Pain in ADPKD

### Risk Factors of Pain in ADPKD

Age and female sex have been associated with pain severity in ADPKD.^14,19^ Combined height-adjusted total liver volume (ht-TLV) and height-adjusted total kidney volume (ht-TKV) were associated with pain in ADPKD (R=0.23, *P* < 0.001), with a more prominent role of ht-TLV (R=0.2, *P* < 0.001) than ht-TKV (R=0.05, *P* = 0.44).^20^ Total kidney volume (TKV) was not associated with pain severity, unless very large (>1000 ml).^6,7,21^ Data from the Observational Study in Patients With Autosomal Dominant Polycystic Kidney Disease study, a large, global, multicenter, prospective observational study evaluating disease progression and patient-reported outcomes in ADPKD, demonstrated that greater baseline ht-TKV correlated with worse physical health-related QOL scores on ADPKD-impact scale (ADPKD-IS; regression coefficient 1.02 [0.65–1.39]).^22^ Numerous studies suggest that elevated BP is associated with reduced pain perception, a phenomenon known as BP-related hypoalgesia,^23^ as an inverse relationship exists between BP and pain sensitivity.^24–26^ However, chronic pain can predispose patients to hypertension.^27^ Central obesity and elevated body mass index have also been associated with increased pain, independent of TKV and total liver volume, possibly due to musculoskeletal causes.^28^ Chronic inflammation, which lowers the pain threshold and exacerbates pain,^29^ is particularly relevant in early stages of ADPKD.^30^

## Acute Versus Chronic Pain in ADPKD

The kidney is innervated by sympathetic, parasympathetic, and afferent fibers (Supplemental Figure 1).^31^ Visceral pain originates from the kidneys and/or liver, while somatic pain arises from the muscles, tendons, and connective tissues.^32^

### Acute Pain in ADPKD

#### Cyst Hemorrhage

Kidney cyst hemorrhage is often seen on CT scans as a high-density mass-like area.^33,34^ Cyst hemorrhage typically presents with sharp, localized pain rather than hematuria, as most distended cysts do not connect with the collecting system.^35^ When gross hematuria occurs, clot passage may lead to renal colic.^36,37^ Most episodes resolve within a week. When hematuria persists, further investigation is required.^14,38^

#### Cyst Infection

Diagnostic criteria for cyst infections include fever, abdominal pain, elevated inflammatory markers, and exclusion of intracystic bleeding on CT.^39,40^ Contrast CT may show thickened cyst walls, heterogeneous contents, and perinephric stranding. Positron emission tomography-CT shows higher 18F-fluorodeoxyglucose uptake in cyst walls compared with the surrounding parenchyma.^41^ A definitive diagnosis requires cyst aspiration, which reveals neutrophils and/or microorganisms.^41^ Management includes urine and blood cultures, followed by antibiotic therapy with a 4- to 6-week regimen of lipid-soluble antibiotics, such as fluoroquinolone or trimethoprim-sulfamethoxazole, which are preferred because they achieve better penetration into cysts and higher intracystic concentration.^42,43^ Hepatic cyst infections also occur, sometimes requiring hospitalization and aspiration.^44^

#### Nephrolithiasis

Nephrolithiasis present as sudden waxing and waning flank pain radiating to the groin.^45,46^ It is commonly precipitated by metabolic factors, such as low urine pH, hypocitraturia, and hyperuricosuria, and anatomical factors, urinary stasis from cyst compression, and distortion of the renal collecting system.^35,47^ Patients with ADPKD with normal kidney function who had a TKV ≥500 ml had a significantly higher prevalence of nephrolithiasis (odds ratio, 6.30 [1.62–24.46]; *P* = 0.008).^35,47^ Noncontrast CT scan is the preferred diagnostic tool.^48^ Stones in the renal pelvis cause dull flank pain, which intensifies as the stone moves through the ureters.^49^ Pain arises from increased intraluminal pressure, PG production, and nerve stretching.^49^ Management starts with hydration, analgesics, and ureteric stent placement when necessary. Potassium citrate is recommended for those with low urinary citrate.^35^ While alpha-blockers such as tamsulosin are commonly used as medical expulsive therapy to facilitate distal ureteral stone passage and reduce pain duration in the general population,^50,51^ their role in ADPKD remains unclear; however, treatment generally follows standard nephrolithiasis management guidelines.^35^ Complicated cases may necessitate extracorporeal shock wave lithotripsy, percutaneous nephrolithotomy, and flexible ureteroscopy.^3,52,53^

### Chronic Abdominal or Flank Pain

Chronic pain typically lasts beyond 4–6 weeks, although some experts use a 3-month threshold.^54,55^ *Post hoc* analysis of the Tolvaptan Efficacy and Safety in Management of Autosomal Dominant Polycystic Kidney Disease and its Outcomes (TEMPO) 3:4 trial indicates that history of pain was present in 50.9% of patients.^56^ Repeated irritation of splanchnic nerves lowers the pain threshold (peripheral sensitization), increasing pain levels.^57^ Over time, recurrent pain episodes may lead to central sensitization, where the nervous system remains hyperreactive, amplifying pain signals even without a stimulus.^57,58^

Chronic abdominal pain is typically poorly localized pain and worsens with movement.^59^ Polycystic liver disease (PLD)^60^ also contributes to abdominal discomfort and early satiety due to progressive hepatomegaly.^61^ In addition, abdominal distention from cyst enlargement can alter posture, causing lumbar lordosis,^10^ and accelerates spinal degeneration, which exacerbates mechanical back pain over time.^62^

## Tools and Techniques for Assessing Pain

The Standardized Outcomes in Nephrology—Polycystic Kidney Disease project has recognized pain in ADPKD as a core outcome for clinical trials,^63,64^ yet its assessment remains inconsistent in practice.^65,66^ A systemic review indicated that only 16 of 68 randomized control trials measured pain as an outcome in ADPKD.^65^ Commonly used tools, such as the visual analog scale (VAS), numeric rating scale, and the ADPKD-specific Assessment Tool, assess pain severity but fail to differentiate kidney-related pain from other sources.^65^

The ADPKD-IS was developed to assess disease burden across all CKD stages, covering physical, emotional, and fatigue domains through 14 core items.^15^ Physical impact assesses daily limitations, emotional impact reflects anxiety and fear of disease progression, and fatigue captures exhaustion. Low scores are more frequent in early CKD, while higher scores indicate greater disease burden and correlate with disease progression.^15^ Although 42% and 77% of patients reported the lowest possible score for each item on the ADPKD-IS, only 1%–6% reported the highest possible score. ADPKD-IS is useful in clinical trials and practice, providing a sensitive measure of disease progression and response to treatments such as tolvaptan.^15^

The ADPKD-Pain and Discomfort Scale (ADPKD-PDS) was designed using global patient input to capture ADPKD-specific pain patterns.^67^ Unlike generic pain scales, ADPKD-PDS identifies three key pain types: chronic dull kidney pain, severe acute kidney pain, and fullness/discomfort. The final version includes 20 items assessing pain intensity and impact on physical activity, social life, and well-being.^65,67^ Between 22% and 70% of participants reported the lowest possible score, while 10% reported the highest item score.^67^ Higher pain scores correlate with advanced CKD stages, lower physical health, and greater disease burden. ADPKD-PDS provides a standardized method to measure pain in clinical trials and practice and better reports pain in ADPKD.^67^

Validated patient-reported outcome measures such as the ADPKD-PDS and ADPKD-IS were developed using qualitative input from patient interviews and focus groups across multiple countries to systematically capture pain severity, patterns, and impact on daily life. These tools indirectly emphasize the importance of pain management by establishing pain as a core patient-reported outcome in ADPKD, although they do not directly evaluate pain management strategies or treatment effectiveness.^15,67^ A systematic review identified 26 different measures used to assess pain in ADPKD, many lacking validation or patient-centered design, highlighting the need for standardized, psychometrically validated instruments.^65^

## Pain and QOL

Pain in ADPKD is a frequent symptom that profoundly affects both QOL and psychologic well-being. Studies have shown that pain leads to significant physical limitations and emotional distress.^21^ This pain, independent of kidney size or function, is associated with reduced physical, emotional, and social functioning and is often underestimated by health care providers.^68^ Furthermore, the psychologic burden of ADPKD extends beyond pain alone as patients experience anxiety and depression as they cope with uncertainty about disease progression, guilt from passing the condition to their children, and fear of organ failure.^69,70^ Symptom burden in PLD can also significantly reduce QOL, particularly affecting mental health and emotional well-being.^71^ If left unaddressed, these psychosocial challenges can lead to long-term socioeconomic consequences, including difficulties in career planning, financial decision making, and family dynamics, further diminishing QOL.^72^ Routine monitoring of psychosocial well-being is recommended over disease course. Clinical guidelines recommend a multidisciplinary approach that includes psychologic support, self-management education, and social services to address the emotional and practical challenges of ADPKD. Providers are encouraged to collaborate with psychologists or psychiatrists, monitor mental health, and integrate pharmacologic and nonpharmacologic interventions.^35^ Early identification and treatment of psychosocial distress are critical to improving well-being and long-term outcomes.^35,73^

### Case Presentation Continued: Stepwise Pain Management

#### Celiac Plexus Block

On follow-up, the pain was chronically at 8/10 despite taking bupropion 150 mg daily, gabapentin 100 mg twice daily, and hydrocodone-acetaminophen 10–325 as needed. Owing to gabapentin-induced palpitations, she was switched to pregabalin 150 mg nightly. To ascertain that her pain is visceral in origin, a celiac plexus block was recommended. She received 3 ml bupivacaine-epinephrine (0.5%), 3 ml lidocaine (20 mg/ml), 10 mg dexamethasone, and 100 mcg clonidine. She experienced significant pain relief for 2 weeks, followed, however, by recurrence of mild right flank soreness.

#### Renal Denervation

At age 30 years, 8 months later, her pain remained uncontrolled, requiring hydrocodone-acetaminophen (10–325 mg) every 6 hours. A CT angiogram revealed three right renal arteries without renal artery stenosis, aneurysms, or fibromuscular dysplasia, but her renal artery anatomy suggested a limited potential benefit from renal denervation. She elected to undergo sequential ablation of two right renal arteries. The procedure, performed under general anesthesia through the right femoral artery, used a cumulative skin dose of 86.66 mGy and a dose area product of 8.81 Gy-cm^2^. Her pain eventually relapsed despite temporary resolution of pain for 4–6 weeks.

#### Spinal Cord Stimulator

Given persistence of pain, a 5-day temporary trial for spinal cord stimulator was recommended but failed to provide relief and was removed. Targeted embolization was considered but is not routinely performed in the United States. With nonsurgical options exhausted, right nephrectomy was considered. Nuclear medicine scan revealed split kidney function of 76% (left, minimally cystic) and 24% (right, severely cystic).

#### Nephrectomy

Repeat magnetic resonance imaging abdomen showed stable kidney volumes and a ht-TLV of 1656.8 ml. Her case was discussed among nephrology, pain medicine, surgery, and interventional radiology, and a shared decision was made to pursue right open nephrectomy with left partial hepatectomy and liver cyst fenestration (Supplemental Figure 2).

## Management of Chronic Pain in ADPKD

Refractory pain in ADPKD should be managed by a multidisciplinary team of the nephrologist, pain specialist, radiologist, psychologist or psychiatrist, and urologist to provide personalized treatments (Figure 2).^74^

Figure 2**Management of acute and chronic pain in ADPKD.** Management diagram for acute (A) and chronic (B) pain in ADPKD. This figure outlines a comprehensive approach to pain management in ADPKD, addressing both acute and chronic pain. Acute pain, often due to cyst hemorrhage, UTI, or kidney stones, is initially managed with supportive care, hydration, and analgesics (acetaminophen, NSAIDs, or opioids if necessary). More severe cases may require antibiotics, drainage, stone removal, or other interventions. Chronic pain (lasting >3 months) requires screening for depression and assessing its relation to kidney function. First-line treatment includes nonpharmacologic strategies (hydration, exercise, weight management, and physical therapy), followed by a stepwise pharmacologic approach, including acetaminophen, NSAIDs, and adjuvant therapies (gabapentin, pregabalin, or TCAs). Refractory pain may require minimally invasive procedures (cyst aspiration, sclerotherapy, and celiac plexus blockade), and in severe cases, invasive options such as renal denervation, arterial embolization, nephrectomy, or transplantation may be considered. ADPKD, autosomal dominant polycystic kidney disease; CT, computed tomography; NSAID, nonsteroidal anti-inflammatory drugs; TCA, tricyclic antidepressant; TENS, transcutaneous electrical nerve stimulation; UCx, urine culture; US, ultrasound; UTI, urinary tract infection.

**Figure:**
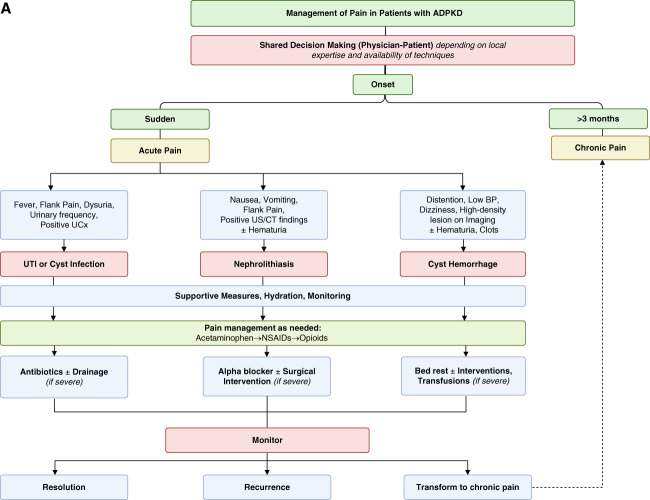

**Figure:**
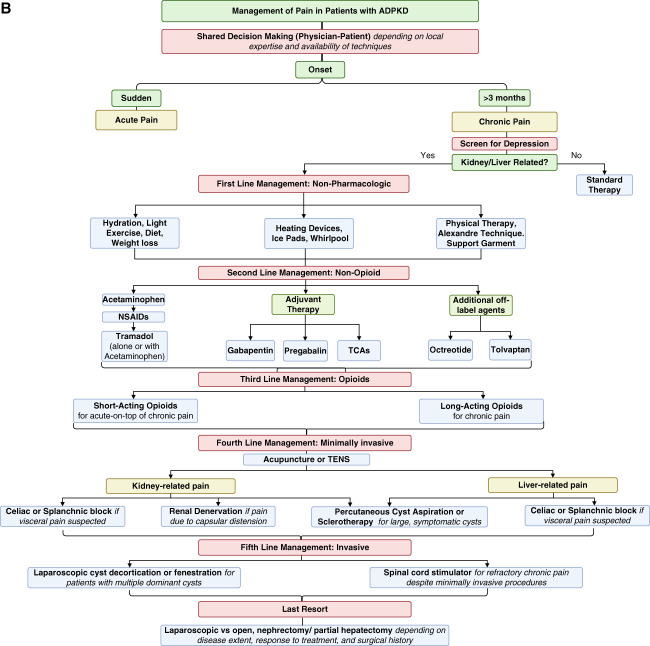

### Nonpharmacologic Management

#### Weight Loss

Weight loss may improve pain perception in fibromyalgia,^75^ osteoarthritis,^76^ and migraine.^77^ It was also associated with decreased cystic growth, where reductions in kidney growth correlated with loss of body weight and visceral adiposity, independent of the dietary regimen used (daily caloric restriction or intermittent fasting).^78^ Even modest weight loss can positively affect back pain. Adequate hydration is recommended for pain management,^28^ although its role in cystic growth and pain modulation remains uncertain due to limited evidence.^79,80^

#### Behavioral Modifications

Physiotherapy, heating or cold patches, and whirlpools may be useful for chronic ADPKD-related pain.^55,81^ The Alexander Technique is an individualized method aimed at improving postural tone and neuromuscular coordination by helping individuals recognize and change habitual patterns of muscle tension and poor movement. It involves guided verbal instruction and hands-on feedback to reduce unnecessary tension with the goal of improving musculoskeletal function and reducing pain.^82^ Despite being commonly used in ADPKD, there are no controlled studies to evaluate its efficacy.^62,82^

#### Acupuncture and TENS

Acupuncture and TENS can both improve ADPKD pain refractory to nonpharmacologic treatments.^62^ TENS, a noninvasive inexpensive approach, uses electric current to the skin to activate nerves within specific regions and inhibit central perception of pain.^83^ TENS was shown to reduce urolithiasis-related pain, with over 50% decrease in VAS scores (85.7±10.5 to 33.3±16.0 mm, *P* < 0.01).^84^

### Pharmacologic Treatment of Pain in ADPKD

#### Acetaminophen

Acetaminophen is endorsed by the National Kidney Foundation as the first analgesic of choice for patients with mild-moderate pain and preexisting kidney disease^62,85^ Acetaminophen should be used with caution in patients with hepatic impairment, chronic alcohol use, or patients on tolvaptan due to the increased risk for hepatotoxicity (Table 1).^86^

**Table 1:** Dosing of the different medications used for pain in autosomal dominant polycystic kidney disease

Medication	Mode of Action	Dosing	Side Effects	Clearance by Dialysis
Acetaminophen^87,88^	Inhibit PG synthesis	CrCl: 10–50 ml/min: 650 mg Q 6 h CrCl <10: 650 mg Q 8 h Max dose 4000 mg	Liver injury, minimal GI side effects	Dialyzed by hemodialysis, not by PD
NSAIDs^87,88^	Inhibits COX-1 and COX-2	200–400 Q 4–6 h Max dose 3200 mg	GI bleed, renal damage	Poorly dialyzed
**Adjuvant analgesics^87,88^**				
Gabapentin	Modulates calcium channels	CrCl: 50–79: 1800 mg CrCl: 30–49: 900 mg CrCl: 15–29: 600 mg CrCl <15: 300 mg^a^	Dizziness and sedation	Possibly dialyzed
Pregabalin	Modulates calcium channels	CrCl: 30–60: 300 mg daily CrCl: 15–30: 150 mg daily CrCl <15: 75 mg daily	Dizziness and sedation	Dialyzed
Duloxetine^87^	Inhibits reuptake of NE and serotonin	Starting with 20 mg daily Max dose 60 mg daily (no dose adjustments needed for kidney function) Avoid use if CrCl <30 (limited data)	Nausea, dry mouth, fatigue, constipation, dizziness	Poorly dialyzed
**TCAs^87,88^**				
Amitriptyline	Inhibits reuptake of NE and serotonin	10 mg daily at night Max dose 150 mg	Dry mouth, constipation, urinary retention, cardiac toxicity	Poorly dialyzed
**Short acting opioids^11,89,90^**				
Oxycodone	Bind to opioid receptors	2.5–5 mg Q 12 h	Constipation, nausea, drowsiness, risk of misuse	Poorly dialyzed
Tramadol	Bind to opioid receptors	Starting from 50 mg Q 12 h to max 200 mg Q 12 h	Constipation, nausea, drowsiness, risk of misuse	Dialyzed
Hydromorphone	Bind to opioid receptors	2.5–5 mg Q 6–8 h	Constipation, nausea, drowsiness, risk of misuse	Dialyzed
Fentanyl	Bind to opioid receptors	Patch, starting from 12 mcg/h	Constipation, nausea, drowsiness, risk of misuse	Poorly dialyzed
**Long-acting opioids^11,89^**				
Methadone	Bind to opioid receptors	1–2 mg every day or every other day	Constipation, nausea, drowsiness, risk of misuse	Poorly dialyzed
Buprenorphine	Bind to opioid receptors	75 mcg orally daily for 4 d then increase to 150 mcg Q 12 Patch: 5 mcg/h once weekly	Constipation, nausea, drowsiness, risk of misuse	Poorly dialyzed

ADPKD, autosomal dominant polycystic kidney disease; CrCl, creatinine clearance; COX-1, cyclooxygenase-1; COX-2, cyclooxygenase-2; GI, gastrointestinal; NSAID, nonsteroidal anti-inflammatory drugs; PD, peritoneal dialysis; Q, every; TCA, tricyclic antidepressants.

a Only short acting gabapentin (Neurontin) can be given if creatinine clearance <30.

#### Nonsteroidal Anti-Inflammatory Drugs

Nonsteroidal anti-inflammatory drug use in ADPKD is approached with caution due to the risk of nephrotoxicity, hypertension,^54^ and hematuria.^91^

#### Analgesic Adjuvants

Analgesic adjuvants have been previously studied for chronic back pain.^92^ Analgesic adjuvants such as gabapentin pregabalin and amitriptyline are commonly used in ADPKD, despite limited large-scale studies.^11,54^ Duloxetine, a serotonin-NE reuptake inhibitor, reduces pain by enhancing central inhibitory pain pathways, although its efficacy in ADPKD remains unexplored.^93^

#### Opioids

Opioids are prescribed when pain is not well controlled by nonopioid agents, but their doses should be adjusted in patients with kidney failure (Table 1). Tramadol, oxycodone, hydromorphone, fentanyl, and buprenorphine are among the commonly used opioids in kidney failure. The strongest evidence supports the utilization of transdermal fentanyl and sustained-release morphine (level II-2), with consideration of oxycodone (level II-3), hydrocodone, and methadone (level III) as other potential options.^11^ Short-acting opioids are initially used for titration and acute pain control.^94^ Once adequate pain control is achieved, patients can be switched to long-acting opioids with the benefits of a lower risk of misuse and decreased dosing frequency.^95^

#### Tolvaptan

Tolvaptan, a vasopressin-2 receptor antagonist, decreases the growth of kidney cysts and decline in kidney function.^55,96–98^ Tolvaptan is associated with reduced pain in patients with ADPKD.^99^ The TEMPO 3:4 trial showed that tolvaptan reduced kidney pain by 36% (hazard ratio, 0.64 [0.48–0.86]) and was associated with a lower incidence of stones and hematuria.^100^ A secondary analysis of TEMPO 3:4 by Casteleijn *et al.* confirmed this benefit across pain severity levels and independent of baseline age, sex, TKV, or history of pain, stones, hematuria, or infections (*P* for interaction > 0.05).^93^ The reduction was partly mediated by fewer renal complications: urinary tract infections (11.1% versus 15.3%; *P* = 0.02), kidney stones (2.2% versus 3.5%; *P* < 0.001), and hematuria (8.0% versus 14.3%; *P* < 0.001).^56^

#### Octreotide

Octreotide is a somatostatin analog that may have beneficial effects in treating neuropathic pain.^101^ Previous clinical trials have shown that it can slow kidney and liver cysts, further alleviating abdominal discomfort and cysts' growth.^60,102^ In a randomized controlled trial, octreotide reduced total liver volume by 71±57 ml compared with placebo over 6 months (*P* < 0.05).^60^ The A Long-Acting somatostatin on DIsease progression in Nephropathy due to autosomal dominant polycystic kidney disease (ALADIN) trial further showed that octreotide-long-acting release reduced kidney volume growth by 96.8 ml at 1 year (*P* = 0.027) and 422.6 ml at 3 years (*P* = 0.002) versus placebo.^102^ Although some analyses have explored its potential to slow progression of kidney function decline, this remains inconclusive and is debated. Somatostatin analogs can be considered in people with ADPKD who have severe symptoms due to massively enlarged kidneys to lower cyst growth rates when no better options are available.^35^

#### Cannabis

Cannabis have been used for chronic pain and muscle spasms,^103^ with its use increasing as legalization expands.^104^ In ADPKD, cannabis is not recommended as it offers no proven clinical benefits and may cause kidney injury.^35,105^

### Noninvasive and Invasive Interventions

#### Spinal Cord Stimulation

Spinal cord stimulation (SCS) involves surgically implanting a device in the epidural space to deliver electrical impulses to the dorsal columns, modulating pain signals before they reach the brain.^11,106^ Although evidence for SCS in ADPKD-related pain is limited, it has been widely used for other chronic pain conditions.^106,107^ To note, some devices may not be magnetic resonance imaging compatible.^108^ In an observational 24-month follow-up of patients who completed a prior multicenter randomized controlled trial on SCS in nonsurgical refractory back pain,^109^ high-frequency SCS for nonsurgical refractory back pain resulted in a 73% reduction in VAS, with 82% of patients achieving at least 50% pain relief at 24 months.^110^

#### Cyst Aspiration and Sclerotherapy

Imaging-guided percutaneous cyst aspiration is a safe, minimally invasive option for treating enlarged kidney or liver cysts.^81,111^ However, simple drainage alone has a high recurrence rate (30%–80%), necessitating the use of sclerosing agents such as ethanol or sodium tetradecyl sulfate (STDS) to improve outcomes.^112,113^ Ethanol sclerotherapy was initially favored due to its low cost, but its failure rate exceeded 30%,^112^ often requiring multiple sessions and increasing risks of pain, fever, intoxication, and retroperitoneal leakage.^113,114^ STDS has a similar success rate to ethanol but is preferred due to its lower pain frequency and severity (pain score 2.1±1.1 versus 3.8±1.2).^114^ Iliuta *et al.* found that STDS foam sclerotherapy reduced TKV by 22% over 14 months, with better pain control and less complications than ethanol. Patients with GFR 60–90 ml/min and large accessible cysts benefited the most.^112^

#### Celiac Plexus Block

Celiac blockade involves the delivery of a neurolytic agent (*i.e*., bupivacaine or ropivacaine) often supplemented with a corticosteroid to mitigate inflammation.^58^ This technique, either alone or followed by major splanchnic nerve block, provides significant pain relief.^35,38^ Radiofrequency ablation or alcohol chemoneurolysis can be used for long-term blockade.^115,116^ Celiac blockade relieved pain in 81.8%, with 36.1% experiencing no recurrence, while radiofrequency ablation of major splanchnic nerve blocks were effective in 87%, leading to opioid cessation in 69.6%.^38^ While celiac blockade targets the visceral pathway, intercostal nerve radiofrequency can interrupt the somatic pathway.^106^

#### Laparoscopic Interventions

Minimally invasive laparoscopic procedures have gained popularity for treating symptomatic kidney and liver cysts, significantly reducing pain postoperatively.^117^ Laparoscopic cyst marsupialization, fenestration, or decortication (cyst deroofing) involve removing the cyst wall and draining its contents, typically performed in patients with severe pain,^62,118^ large cysts (>5 cm), or multiple dominant cysts (>4 cm each).^62,74^ A meta-analysis found that laparoscopic fenestration in patients with symptomatic liver cysts effectively relieves symptoms in 90.2% of patients, with low recurrence (9.6%) and reintervention rates (7.1%), but PLD patients face higher recurrence (33.7%) and complication rates (29.3%).^119^

#### Renal Denervation

Endovascular renal denervation has shown mixed outcomes in ADPKD, with borderline significant pain reduction in a study of five patients unresponsive to celiac plexus block.^38^ A study involving 12 pediatric patients with ADPKD who underwent laparoscopic renal denervation reported pain relief after 25.5 months.^120^ Denervation can be performed through thoracoscopy, laparoscopy, transluminal radiofrequency, or open surgery.^115,121–123^ Complete verve removal is crucial as partial removal leads to poor outcomes.^11,122^ Sympatho-splanchnicectomy is another surgical technique used in patients with chronic abdominal pain, including ADPKD pain.^122,124^

#### Transcatheter Arterial Embolization

Transcatheter arterial embolization (TAE) has been used instead of nephrectomy while preparing for kidney transplantation in ADPKD.^125,126^ TAE in ADPKD was first suggested and mainly performed in Japan. It improved symptoms and showed 46%–54% reduction in TKV over 12 months.^127–129^ TAE is also a good option for patients with PLD.^9^

#### Unilateral or Bilateral Nephrectomy

When all other treatments fail, up to 30% of patients with ADPKD with refractory pain may ultimately require nephrectomy.^6,106,130^ Laparoscopic nephrectomy is the preferred approach due to its lower morbidity and superior outcomes, but open nephrectomy remains necessary for patients with significantly enlarged kidneys.^11^ Studies have demonstrated effective pain relief postnephrectomy in ADPKD.^131–133^ Laparoscopic nephrectomy in nine symptomatic ADPKD patients with kidney failure resulted in complete pain elimination in 100% of cases over 31 months.^131^ Nephrectomy in ADPKD requires special consideration in the transplant setting, as it may accelerate the need for transplantation or dialysis and carries implications for both physical and psychosocial outcomes.^134,135^ Guidelines emphasize the importance of balancing symptom relief with surgical risks and future transplant eligibility, recommending multidisciplinary evaluation to guide timing and appropriateness^35^ Current guidelines emphasize that nephrectomy decisions in ADPKD should consider both clinical and patient-reported outcomes. Pain, early satiety, abdominal distension, and disturbed body image are key factors influencing nephrectomy consideration.^134,135^

#### Partial Hepatectomy

Liver resection is the preferred treatment for severe symptomatic PLD when less invasive options fail. It is performed only after ensuring adequate residual liver function and assessing morbidity and mortality risk.^9,136^ Liver resection with cyst fenestration can improve pain and QOL in patients with severe cases.^137,138^ As a final step, liver transplant can be performed for massive PLD with high symptom burden.^35^

### Case Presentation Continued: Postsurgical Outcomes

#### Postoperative Course

**Figure 3:**
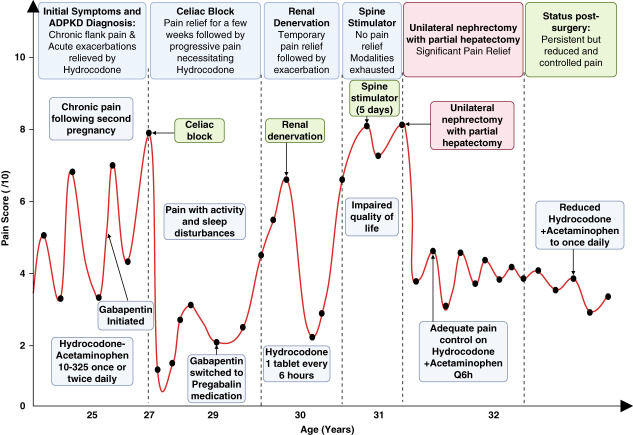
**Evolution of the pain experienced by our patient with age and interventions.** This figure illustrates the progression of pain and the various treatment interventions experienced by our 29-year-old patient diagnosed with ADPKD at the age of 11 years. Symptoms started to develop and exacerbate after her second pregnancy. Initially, her chronic right-sided flank pain was intermittently managed with acetaminophen-hydrocodone and required adjuvant therapy throughout the following years. Gabapentin was initiated after repeated hospitalizations for acute pain, but a switch to pregabalin was necessary due to side effects and increasing pain. A celiac plexus block provided temporary relief, but the pain soon returned, prompting a renal denervation procedure, which also resulted in short-term improvement followed by a recurrence of pain. A spinal cord stimulator trial was attempted but failed to offer significant pain relief. Ultimately, after exhausting noninvasive and semi-invasive options, the patient underwent a right nephrectomy combined with a hepatectomy and liver cyst fenestration which provided substantial and sustained pain relief, improving her QOL and reducing the need for narcotics to manage pain. Q, every; QOL, quality of life.

One month postoperatively, the patient reported an 80%–85% reduction in pain, requiring hydrocodone (once daily, down from every 6 hours), methocarbamol as needed, and pregabalin. She also reported an overall improvement in QOL, as reflected by a reduction in her ADPKD-IS from 73 to 58 (post-operatively). Six months postoperatively, she developed incisional hernias, requiring abdominal wall reconstruction with retrorectus mesh repair. One year after her right nephrectomy (age 32 years), she noted significant improvement in right flank pain, although still present, with intermittent dull left-sided flank pain (Figure 3).

## Conclusion

This review highlights abdominal and flank pain as a common manifestation of ADPKD. Pain can occur either directly due to kidney and/or liver cyst expansion or indirectly due to ADPKD complications such as hemorrhage, infection, or nephrolithiasis.^5,6,11^ Understanding the onset, character and type of pain, whether acute or chronic, is crucial for individualized management.^11,139,140^ Managing ADPKD-related pain requires a multidisciplinary approach, using a stepwise management plan including nonpharmacologic, pharmacologic, minimally invasive therapies, and surgical modalities.^7,74^ However, it is important to recognize that pain management must be individualized, and strategies effective in one case may not be generalizable to all patients with ADPKD. We acknowledge that the pain management approach described here reflects a personalized decision based on patient-specific factors and clinical context, introducing potential selection bias. Although nephrectomy provided symptom relief in this case, further research and clinical trials are needed to evaluate the effectiveness, risks, and broader applicability of such interventions.

## Supplementary Material

## Supplemental Material

Supplemental Figure 1. The innervation of the kidney along with the location of the different pain-blocking techniques.

Supplemental Figure 2. Images of the right cystic kidney post nephrectomy (A) and resected liver section (B) post partial hepatectomy.
