# Supplementary material for: Pain Management in Autosomal Dominant Polycystic Kidney Disease: Clinical Challenges and a Stepwise Algorithmic Approach
Source: Kidney360. 2025 Jul 7;6(9):1618–31. doi: 10.34067/KID.0000000907 (PMC12483046; doi:10.34067/KID.0000000907)
Supplement: Supplementary file 1 [file kidney360-6-01618-s001.pdf]

ASN American Society of Nephrology · JASN Journal of the American Society of Nephrology · KIDNEY360 Accessing Our World From Every Angle · CJASN Clinical Journal of the American Society of Nephrology

ASN Journal Disclosure Form

As per ASN journal policy, I have disclosed any financial relationships or commitments I have held in the past 36 months as included below. I have listed my Current Employer below to indicate there is a relationship requiring disclosure. If no relationship exists, my Current Employer is not listed.

L. Baker reports the following:
Employer: Mayo Clinic; and Ownership Interest: BAX: Baxter International; OM: Outset Medical; DIS: Walt Disney Company; NVDA: Nvidia; TSLA: Tesla.

I understand that the information above will be published within the journal article, if accepted, and that failure to comply and/or to accurately and completely report the potential financial conflicts of interest could lead to the following: 1) Prior to publication, article rejection, or 2) Post-publication, sanctions ranging from, but not limited to, issuing a correction, reporting the inaccurate information to the authors' institution, banning authors from submitting work to ASN journals for varying lengths of time, and/or retraction of the published work.

Name: Lyle Wesley Baker
Manuscript ID: K360-2025-000338R1
Manuscript Title: Pain Management in Autosomal Dominant Polycystic Kidney Disease: Clinical Challenges and a Stepwise Algorithmic Approach
Date of Completion: May 8, 2025
Disclosure Updated Date: May 8, 2025

ASN
American Society of Nephrology
JASN
Journal of the American Society of Nephrology
KIDNEY360
Accessing Our World From Every Angle
CJASN
Clinical Journal of the American Society of Nephrology

ASN Journal Disclosure Form

As per ASN journal policy, I have disclosed any financial relationships or commitments I have held in the past 36 months as included below. I have listed my Current Employer below to indicate there is a relationship requiring disclosure. If no relationship exists, my Current Employer is not listed.

A. Borghol reports the following:
Employer: Mayo Clinic Florida

I understand that the information above will be published within the journal article, if accepted, and that failure to comply and/or to accurately and completely report the potential financial conflicts of interest could lead to the following: 1) Prior to publication, article rejection, or 2) Post-publication, sanctions ranging from, but not limited to, issuing a correction, reporting the inaccurate information to the authors' institution, banning authors from submitting work to ASN journals for varying lengths of time, and/or retraction of the published work.

Name: Abdul Hamid Borghol
Manuscript ID: K360-2025-000338R1
Manuscript Title: Pain Management in Autosomal Dominant Polycystic Kidney Disease: Clinical Challenges and a Stepwise Algorithmic Approach
Date of Completion: May 8, 2025
Disclosure Updated Date: May 8, 2025

ASN American Society of Nephrology · JASN Journal of the American Society of Nephrology · KIDNEY360 Accessing Our World From Every Angle · CJASN Clinical Journal of the American Society of Nephrology

ASN Journal Disclosure Form

As per ASN journal policy, I have disclosed any financial relationships or commitments I have held in the past 36 months as included below. I have listed my Current Employer below to indicate there is a relationship requiring disclosure. If no relationship exists, my Current Employer is not listed.

M. Bou Antoun has nothing to disclose.

I understand that the information above will be published within the journal article, if accepted, and that failure to comply and/or to accurately and completely report the potential financial conflicts of interest could lead to the following: 1) Prior to publication, article rejection, or 2) Post-publication, sanctions ranging from, but not limited to, issuing a correction, reporting the inaccurate information to the authors' institution, banning authors from submitting work to ASN journals for varying lengths of time, and/or retraction of the published work.

Name: Marie Therese Bou Antoun
Manuscript ID: K360-2025-000338R1
Manuscript Title: Pain Management in Autosomal Dominant Polycystic Kidney Disease: Clinical Challenges and a Stepwise Algorithmic Approach
Date of Completion: May 13, 2025
Disclosure Updated Date: May 9, 2025

ASN
American Society of Nephrology
JASN
Journal of the American Society of Nephrology
KIDNEY360
Accessing Our World From Every Angle
CJASN
Clinical Journal of the American Society of Nephrology

ASN Journal Disclosure Form

As per ASN journal policy, I have disclosed any financial relationships or commitments I have held in the past 36 months as included below. I have listed my Current Employer below to indicate there is a relationship requiring disclosure. If no relationship exists, my Current Employer is not listed.

F. Chebib reports the following:
Employer: Mayo Clinic; Research Funding: Research grant- Otsuka pharmaceuticals; Natera; Regulus; Vertex; and Patents or Royalties: Patent no US20200368191A1.

I understand that the information above will be published within the journal article, if accepted, and that failure to comply and/or to accurately and completely report the potential financial conflicts of interest could lead to the following: 1) Prior to publication, article rejection, or 2) Post-publication, sanctions ranging from, but not limited to, issuing a correction, reporting the inaccurate information to the authors' institution, banning authors from submitting work to ASN journals for varying lengths of time, and/or retraction of the published work.

Name: Fouad T. Chebib
Manuscript ID: K360-2025-000338R1
Manuscript Title: Pain Management in Autosomal Dominant Polycystic Kidney Disease: Clinical Challenges and a Stepwise Algorithmic Approach
Date of Completion: May 9, 2025
Disclosure Updated Date: May 8, 2025

ASN American Society of Nephrology
JASN Journal of the American Society of Nephrology
KIDNEY360 Accessing Our World From Every Angle
CJASN Clinical Journal of the American Society of Nephrology

ASN Journal Disclosure Form

As per ASN journal policy, I have disclosed any financial relationships or commitments I have held in the past 36 months as included below. I have listed my Current Employer below to indicate there is a relationship requiring disclosure. If no relationship exists, my Current Employer is not listed.

A. Ghanem reports the following:
Employer: Mayo Clinic

I understand that the information above will be published within the journal article, if accepted, and that failure to comply and/or to accurately and completely report the potential financial conflicts of interest could lead to the following: 1) Prior to publication, article rejection, or 2) Post-publication, sanctions ranging from, but not limited to, issuing a correction, reporting the inaccurate information to the authors' institution, banning authors from submitting work to ASN journals for varying lengths of time, and/or retraction of the published work.

Name: Ahmad Ghanem
Manuscript ID: K360-2025-000338R1
Manuscript Title: Pain Management in Autosomal Dominant Polycystic Kidney Disease: Clinical Challenges and a Stepwise Algorithmic Approach
Date of Completion: May 8, 2025
Disclosure Updated Date: May 11, 2024

ASN American Society of Nephrology
JASN Journal of the American Society of Nephrology
KIDNEY360 Accessing Our World From Every Angle
CJASN Clinical Journal of the American Society of Nephrology

ASN Journal Disclosure Form

As per ASN journal policy, I have disclosed any financial relationships or commitments I have held in the past 36 months as included below. I have listed my Current Employer below to indicate there is a relationship requiring disclosure. If no relationship exists, my Current Employer is not listed.

S. Gupta has nothing to disclose.

I understand that the information above will be published within the journal article, if accepted, and that failure to comply and/or to accurately and completely report the potential financial conflicts of interest could lead to the following: 1) Prior to publication, article rejection, or 2) Post-publication, sanctions ranging from, but not limited to, issuing a correction, reporting the inaccurate information to the authors' institution, banning authors from submitting work to ASN journals for varying lengths of time, and/or retraction of the published work.

Name: Sahil Gupta
Manuscript ID: K360-2025-000338R1
Manuscript Title: Pain Management in Autosomal Dominant Polycystic Kidney Disease: Clinical Challenges and a Stepwise Algorithmic Approach
Date of Completion: May 9, 2025
Disclosure Updated Date: May 9, 2025

ASN American Society of Nephrology JASN Journal of the American Society of Nephrology KIDNEY360 Accessing Our World From Every Angle CJASN Clinical Journal of the American Society of Nephrology

ASN Journal Disclosure Form

As per ASN journal policy, I have disclosed any financial relationships or commitments I have held in the past 36 months as included below. I have listed my Current Employer below to indicate there is a relationship requiring disclosure. If no relationship exists, my Current Employer is not listed.

M. Hassanein has nothing to disclose.

I understand that the information above will be published within the journal article, if accepted, and that failure to comply and/or to accurately and completely report the potential financial conflicts of interest could lead to the following: 1) Prior to publication, article rejection, or 2) Post-publication, sanctions ranging from, but not limited to, issuing a correction, reporting the inaccurate information to the authors' institution, banning authors from submitting work to ASN journals for varying lengths of time, and/or retraction of the published work.

Name: Mohamed Hassanein
Manuscript ID: K360-2025-000338R1
Manuscript Title: Pain Management in Autosomal Dominant Polycystic Kidney Disease: Clinical Challenges and a Stepwise Algorithmic Approach
Date of Completion: June 20, 2025
Disclosure Updated Date: June 20, 2025

ASN
American Society of Nephrology
JASN
Journal of the American Society of Nephrology
KIDNEY360
Accessing Our World From Every Angle
CJASN
Clinical Journal of the American Society of Nephrology

ASN Journal Disclosure Form

As per ASN journal policy, I have disclosed any financial relationships or commitments I have held in the past 36 months as included below. I have listed my Current Employer below to indicate there is a relationship requiring disclosure. If no relationship exists, my Current Employer is not listed.

M. Hogan reports the following:
Employer: Mayo Clinic; Consultancy: Otsuka pharmaceuticals; Research Funding: Camurus, Regulus Pharmaceuticals., Reata.; Advisory or Leadership Role: Mayo Clinic Proceedings Quality & Outcomes Journal; No payment; Camurus Pharmaceuticals.; No payment; Sail Bio, No payment.; Glaxo-Smith-Kline, No payment. American Society of Nephrology, no payment. Regulus - no payment. Glaxo Smith Kline no payment.; and Other Interests or Relationships: PKD Foundation;; PKD Disease Outcomes Consortium; American Society of Nephrology;.

I understand that the information above will be published within the journal article, if accepted, and that failure to comply and/or to accurately and completely report the potential financial conflicts of interest could lead to the following: 1) Prior to publication, article rejection, or 2) Post-publication, sanctions ranging from, but not limited to, issuing a correction, reporting the inaccurate information to the authors' institution, banning authors from submitting work to ASN journals for varying lengths of time, and/or retraction of the published work.

Name: Marie C. Hogan
Manuscript ID: K360-2025-000338R1
Manuscript Title: Pain Management in Autosomal Dominant Polycystic Kidney Disease: Clinical Challenges and a Stepwise Algorithmic Approach
Date of Completion: May 9, 2025
Disclosure Updated Date: December 11, 2024

ASN American Society of Nephrology
JASN Journal of the American Society of Nephrology
KIDNEY360 Accessing Our World From Every Angle
CJASN Clinical Journal of the American Society of Nephrology

ASN Journal Disclosure Form

As per ASN journal policy, I have disclosed any financial relationships or commitments I have held in the past 36 months as included below. I have listed my Current Employer below to indicate there is a relationship requiring disclosure. If no relationship exists, my Current Employer is not listed.

C. Hunt reports the following:
Employer: Mayo Clinic; Research Funding: Nevro, Inc; and Advisory or Leadership Role: Board of Directors, member at large - North American Neuromodulation Society and ASRA Pain Medicine; (neither is paid, both are non-profits).

I understand that the information above will be published within the journal article, if accepted, and that failure to comply and/or to accurately and completely report the potential financial conflicts of interest could lead to the following: 1) Prior to publication, article rejection, or 2) Post-publication, sanctions ranging from, but not limited to, issuing a correction, reporting the inaccurate information to the authors' institution, banning authors from submitting work to ASN journals for varying lengths of time, and/or retraction of the published work.

Name: Christine Hunt
Manuscript ID: K360-2025-000338R1
Manuscript Title: Pain Management in Autosomal Dominant Polycystic Kidney Disease: Clinical Challenges and a Stepwise Algorithmic Approach
Date of Completion: June 20, 2025
Disclosure Updated Date: June 20, 2025

ASN
American Society of Nephrology
JASN
Journal of the American Society of Nephrology
KIDNEY360
Accessing Our World From Every Angle
CJASN
Clinical Journal of the American Society of Nephrology

ASN Journal Disclosure Form

As per ASN journal policy, I have disclosed any financial relationships or commitments I have held in the past 36 months as included below. I have listed my Current Employer below to indicate there is a relationship requiring disclosure. If no relationship exists, my Current Employer is not listed.

M. Mao reports the following:
Employer: Mayo Clinic College Of Medicine (Jacksonville)

I understand that the information above will be published within the journal article, if accepted, and that failure to comply and/or to accurately and completely report the potential financial conflicts of interest could lead to the following: 1) Prior to publication, article rejection, or 2) Post-publication, sanctions ranging from, but not limited to, issuing a correction, reporting the inaccurate information to the authors' institution, banning authors from submitting work to ASN journals for varying lengths of time, and/or retraction of the published work.

Name: Michael A. Mao
Manuscript ID: K360-2025-000039R1
Manuscript Title: Multicenter Insights into Peritoneal Dialysis for ADPKD: Role of Cumulative Cystic Organ Volumes in Treatment Complications
Date of Completion: May 13, 2025
Disclosure Updated Date: May 13, 2025

ASN American Society of Nephrology
JASN Journal of the American Society of Nephrology
KIDNEY360 Accessing Our World From Every Angle
CJASN Clinical Journal of the American Society of Nephrology

ASN Journal Disclosure Form

As per ASN journal policy, I have disclosed any financial relationships or commitments I have held in the past 36 months as included below. I have listed my Current Employer below to indicate there is a relationship requiring disclosure. If no relationship exists, my Current Employer is not listed.

S. Mao reports the following:
Employer: Mayo Clinic

I understand that the information above will be published within the journal article, if accepted, and that failure to comply and/or to accurately and completely report the potential financial conflicts of interest could lead to the following: 1) Prior to publication, article rejection, or 2) Post-publication, sanctions ranging from, but not limited to, issuing a correction, reporting the inaccurate information to the authors' institution, banning authors from submitting work to ASN journals for varying lengths of time, and/or retraction of the published work.

Name: Shennen Mao
Manuscript ID: K360-2025-000338R1
Manuscript Title: Pain Management in Autosomal Dominant Polycystic Kidney Disease: Clinical Challenges and a Stepwise Algorithmic Approach
Date of Completion: June 20, 2025
Disclosure Updated Date: December 11, 2024

ASN
American Society of Nephrology
JASN
Journal of the American Society of Nephrology
KIDNEY360
Accessing Our World From Every Angle
CJASN
Clinical Journal of the American Society of Nephrology

ASN Journal Disclosure Form

As per ASN journal policy, I have disclosed any financial relationships or commitments I have held in the past 36 months as included below. I have listed my Current Employer below to indicate there is a relationship requiring disclosure. If no relationship exists, my Current Employer is not listed.

J. Mina reports the following:
Employer: Staten Island University Hospital, Northwell Health

I understand that the information above will be published within the journal article, if accepted, and that failure to comply and/or to accurately and completely report the potential financial conflicts of interest could lead to the following: 1) Prior to publication, article rejection, or 2) Post-publication, sanctions ranging from, but not limited to, issuing a correction, reporting the inaccurate information to the authors' institution, banning authors from submitting work to ASN journals for varying lengths of time, and/or retraction of the published work.

Name: Jonathan Mina
Manuscript ID: K360-2025-000338R1
Manuscript Title: Pain Management in Autosomal Dominant Polycystic Kidney Disease: Clinical Challenges and a Stepwise Algorithmic Approach
Date of Completion: June 20, 2025
Disclosure Updated Date: June 20, 2025

ASN Journal Disclosure Form

As per ASN journal policy, I have disclosed any financial relationships or commitments I have held in the past 36 months as included below. I have listed my Current Employer below to indicate there is a relationship requiring disclosure. If no relationship exists, my Current Employer is not listed.

F. Munairdjy Debeh reports the following:
Employer: Mayo Clinic Florida

I understand that the information above will be published within the journal article, if accepted, and that failure to comply and/or to accurately and completely report the potential financial conflicts of interest could lead to the following: 1) Prior to publication, article rejection, or 2) Post-publication, sanctions ranging from, but not limited to, issuing a correction, reporting the inaccurate information to the authors' institution, banning authors from submitting work to ASN journals for varying lengths of time, and/or retraction of the published work.

Name: Fadi George Munairdjy Debeh
Manuscript ID: K360-2025-000338R1
Manuscript Title: Pain Management in Autosomal Dominant Polycystic Kidney Disease: Clinical Challenges and a Stepwise Algorithmic Approach.
Date of Completion: May 9, 2025
Disclosure Updated Date: May 8, 2025

ASN
American Society of Nephrology
JASN
Journal of the American Society of Nephrology
KIDNEY360
Accessing Our World From Every Angle
CJASN
Clinical Journal of the American Society of Nephrology

ASN Journal Disclosure Form

As per ASN journal policy, I have disclosed any financial relationships or commitments I have held in the past 36 months as included below. I have listed my Current Employer below to indicate there is a relationship requiring disclosure. If no relationship exists, my Current Employer is not listed.

V. Rangarajan has nothing to disclose.

I understand that the information above will be published within the journal article, if accepted, and that failure to comply and/or to accurately and completely report the potential financial conflicts of interest could lead to the following: 1) Prior to publication, article rejection, or 2) Post-publication, sanctions ranging from, but not limited to, issuing a correction, reporting the inaccurate information to the authors' institution, banning authors from submitting work to ASN journals for varying lengths of time, and/or retraction of the published work.

Name: Vineetha Rangarajan
Manuscript ID: K360-2025-000338R1
Manuscript Title: Pain Management in Autosomal Dominant Polycystic Kidney Disease: Clinical Challenges and a Stepwise Algorithmic Approach
Date of Completion: May 14, 2025
Disclosure Updated Date: May 14, 2025
