# Supplementary material for: Pain Management in Autosomal Dominant Polycystic Kidney Disease: Clinical Challenges and a Stepwise Algorithmic Approach
Source: Kidney360. 2025 Jul 7;6(9):1618–31. doi: 10.34067/KID.0000000907 (PMC12483046; doi:10.34067/KID.0000000907)

Supplemental:

References:

1- Bajwa ZH, Gupta S, Warfield CA, Steinman TI: Pain management in polycystic kidney disease. *Kidney Int,* 60**:** 1631-1644, 2001 10.1046/j.1523-1755.2001.00985.x

2- Ammons WS: Renal afferent input to thoracolumbar spinal neurons of the cat. Am J Physiol, 250: R435-443, 1986 10.1152/ajpregu.1986.250.3.R435

3- Casteleijn NF, Visser FW, Drenth JP, Gevers TJ, Groen GJ, Hogan MC, Gansevoort RT, Consortium D: A stepwise approach for effective management of chronic pain in autosomal-dominant polycystic kidney disease. Nephrol Dial Transplant, 29 Suppl 4: iv142-153, 2014 10.1093/ndt/gfu073

4- Ammons WS: Bowditch Lecture. Renal afferent inputs to ascending spinal pathways. Am J Physiol, 262: R165-176, 1992 10.1152/ajpregu.1992.262.2.R165

Supplemental Figure 1: The innervation of the kidney along with the location of the different pain-blocking techniques

Sympathetic fibers (T10–L2) travel via the splanchnic nerves to vascular and smooth muscle structures,[1,2] while parasympathetic fibers (vagus nerve) target the renal pelvis and calyces.[1] Pain signals are transmitted via C-fibers and A-delta fibers, following sympathetic pathways to the spinal cord and thalamus, with referral patterns based on T5–T6 (celiac plexus) and T11–T12 (splanchnic nerves).[3,4] The figure also highlights nerve block interventions-splanchnic nerve block, celiac plexus block, and renal denervation-used for pain management in kidney-related conditions

Supplemental Figure 2: Images of the right cystic kidney post nephrectomy (A) and resected liver section (B) post partial hepatectomy

Supplemental Figure 1: The innervation of the kidney along with the location of the different pain-blocking techniques

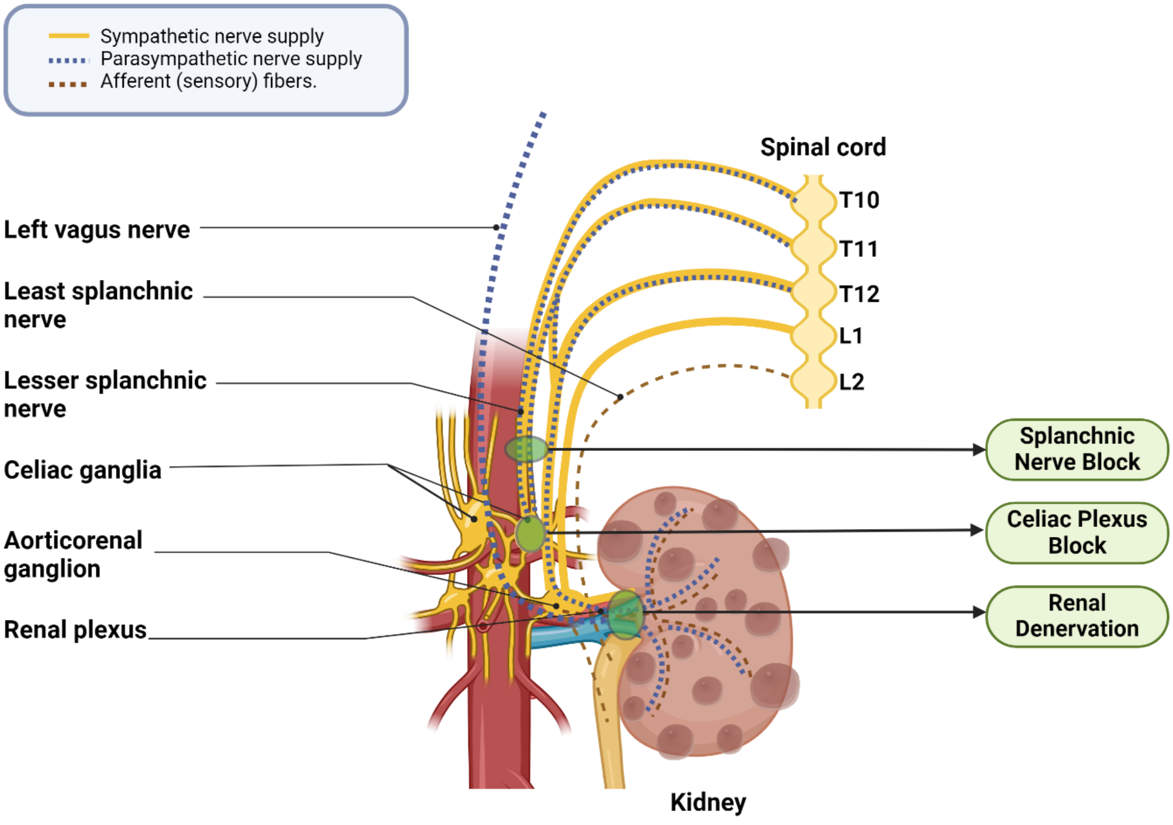

Supplemental Figure 2: Images of the right cystic kidney post nephrectomy (A) and resected liver section (B) post partial hepatectomy.

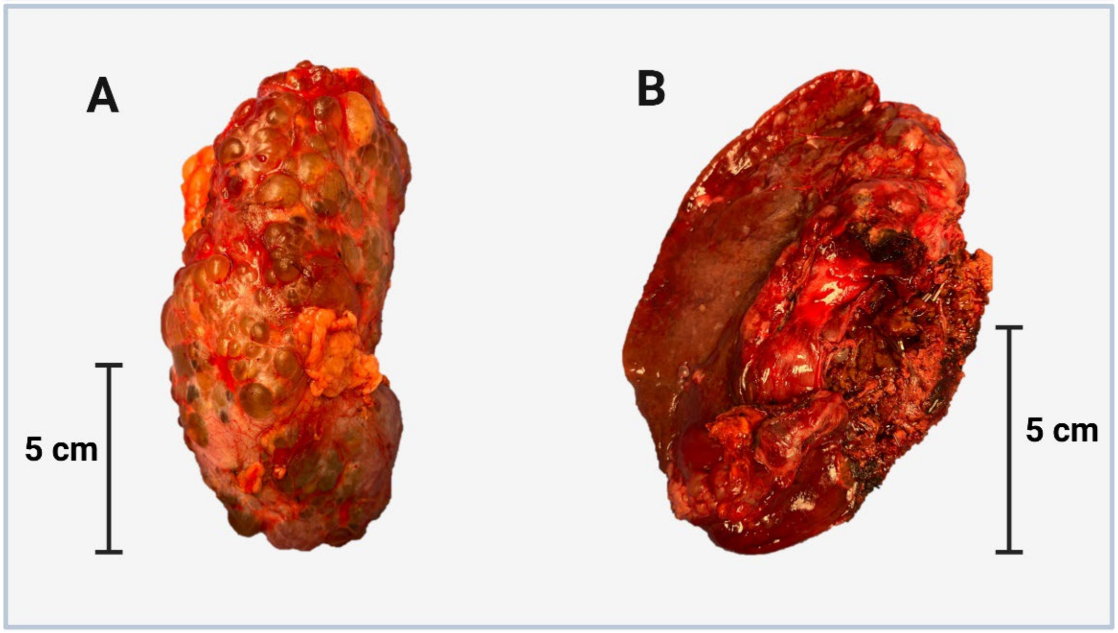

Supplement: Supplementary file 2 [file kidney360-6-01618-s002.pdf]
